# An Overview of Neoplasia in Captive Wild Felids in Southern Italy Zoos

**DOI:** 10.3389/fvets.2022.899481

**Published:** 2022-05-10

**Authors:** Ilaria d'Aquino, Giuseppe Piegari, Silvia Mariagiovanna Casciaro, Francesco Prisco, Guido Rosato, Pasquale Silvestre, Barbara Degli Uberti, Michele Capasso, Piero Laricchiuta, Orlando Paciello, Valeria Russo

**Affiliations:** ^1^Unit of Pathology, Department of Veterinary Medicine and Animal Production, University of Naples Federico II, Naples, Italy; ^2^Centro Regionale per l'Igiene Urbana Veterinaria (CRIUV), Naples, Italy; ^3^Zoo di Napoli, Naples, Italy; ^4^Istituto Zooprofilattico Sperimentale del Mezzogiorno (IZSM), Naples, Italy; ^5^Maitine Zoo, Benevento, Italy; ^6^Fasano Zoo, Brindisi, Italy

**Keywords:** captive felids, neoplasm, zoo, cancer, Southern Italy

## Abstract

The aim of this study was to evaluate the frequency of neoplasms in captive wild felids in Southern Italy zoos over a 13-year period (2008–2021) and to investigate macroscopic and histologic tumor findings in these animals. A total of 24 cases were necropsied, 9 males and 15 females, with age ranging from 6 to 19 years, including 12 tigers (*Panthera tigris*), 7 leopards (*Panthera pardus*), 4 lions (*Panthera leo*), and 1 black jaguar (*Panthera onca*). Diagnosis of neoplasm was made in 14/24 cases (58.3%). Tumors diagnosed were two cholangiocarcinomas, two hemangiosarcomas of the liver, two uterine leiomyomas, a renal adenocarcinoma, an adrenal gland adenoma, a thyroid carcinoma, an oral squamous cell carcinoma, an osteoma, a meningioma, a mesothelioma, an esophageal leiomyosarcoma, a muscoloskeletal leiomyosarcoma and a thyroid adenoma. The malignant and benign tumors were 62.5 and 37.5%, respectively. Among malignant tumors, no metastasis was observed in 50% of cases; in 10% of cases metastasis involved only regional lymph nodes; and distant metastases were found in 40% of cases. Based on our findings, the liver was the most frequent primary tumor site (25%). The high rates of malignant and widely metastatic neoplasms suggest the importance of active monitoring and management of neoplasia in these threatened and endangered species.

## Introduction

Over the last decades, an increasing number of wild animals live in urbanized areas because of zoos and zoological gardens, and they are faced with the need to adapt to the urban ecosystem (1). In this environment, they are highly exposed to pollutants (e.g., chemicals, light, and noise), contaminants in the air, water, and food, and new infections that, in the literature, are reported as predisposing factors of cancer in both humans and animals (2, 3). According to scientific literature, neoplastic diseases are an important cause of morbidity and mortality in several wildlife species (4). It is hypothesized that cancer occurrence is higher in captive animals compared with their wild counterpart because of their longer life expectancy (5, 6). In captive felids, a wide range of different tumors has been described, most of which are reported as single cases (6, 7), and only few studies focused on tumor frequency (8–12). The aim of this study was to evaluate the frequency of neoplasms in captive wild felids in Southern Italy zoos and to investigate macroscopic and histologic tumor findings in these animals.

## Materials and Methods

### Study Design

The present study was structured as an observational retrospective study of captive wild felids in Southern Italy zoos over a 13-year period (2008–2021). All animals were submitted for necropsy to the University of Naples “Federico II” and to the “Istituto Zooprofilattico del Mezzogiorno” (IZSM) of Portici, Southern Italy. The submission forms were collected to obtain information about the species, age, sex, zoological garden where they were housed, and clinical signs. All animals died naturally or were euthanized and a complete necropsy was performed not later than 24 h after death. Inclusion criteria were a complete signalment, post-mortem examination, and at least 3 years of permanence in the submitting zoo. All cases included in this study underwent a complete gross examination performed with our standard necropsy protocol (13). Furthermore, for each assessed animal, representative samples for histopathological examination were collected and evaluated from multiple organs, including the respiratory and gastrointestinal tract, liver, pancreas, urogenital tract, heart, hematopoietic organs, musculoskeletal system, endocrine organs, peripheral as well as central nervous system. Briefly, samples were fixed in 10% buffered formalin and embedded in paraffin; 3 μm-thick sections were cut and stained with Haematoxylin and Eosin. Immunohistochemistry examination was performed in 3 cases. Paraffin-embedded samples were sectioned, dewaxed with xylene, hydrated, and irradiated in a microwave oven (maximum power, 800 W) in tris ethylenedi-amine tetraacetic acid buffer (EDTA; 10 mM Tris base, 1 mM EDTA solution, 0.05% Tween 20) pH 9.0, for 10 min. The peroxidase activity was inhibited by immersing the slides in hydrogen peroxide 0.3% in absolute methanol for 20 min. The sections were incubated overnight at 4 °C with primary antibodies: vimentin (DAKO, clone 9, IR630, Denmark), α-smooth muscle actin (DAKO, clone 1A4, M0851, Carpinteria, CA), synaptophysin (DAKO, clone DAK-SYNAP, M7315, Denmark), S-100 (DAKO, Polyclonal, IS504, Denmark) and cytokeratin (DAKO, Clone AE1/AE3, GA053, Carpinteria, CA). The slides were washed with PBS, then incubated with biotinylated secondary anti-body, and labeled with streptavidin biotin for 30 min at room temperature, followed by incubation with streptavidin conjugated to horseradish peroxidase (LSAB Kit, Dako Ctomation, Glostrup, Denmark). The reaction was revealed by diaminobenzidine treatment (Dako Cytomation, Denmark), and finally the sections were counterstained with Mayer's hematoxylin (14).

### Statistical Analysis

Frequencies were calculated for each categorical variable included in the study. Necropsy neoplasia rate (NNR) was calculated assessing the ratio between the number of animals with neoplasia and the total of cases included in the study. All rates were reported as percentages. The Chi-square test was used to assess differences of age between animals with neoplasia and animals without neoplasia. The statistical analysis was performed using SPSS® (ver. 13).

## Results

**Figure 1:**
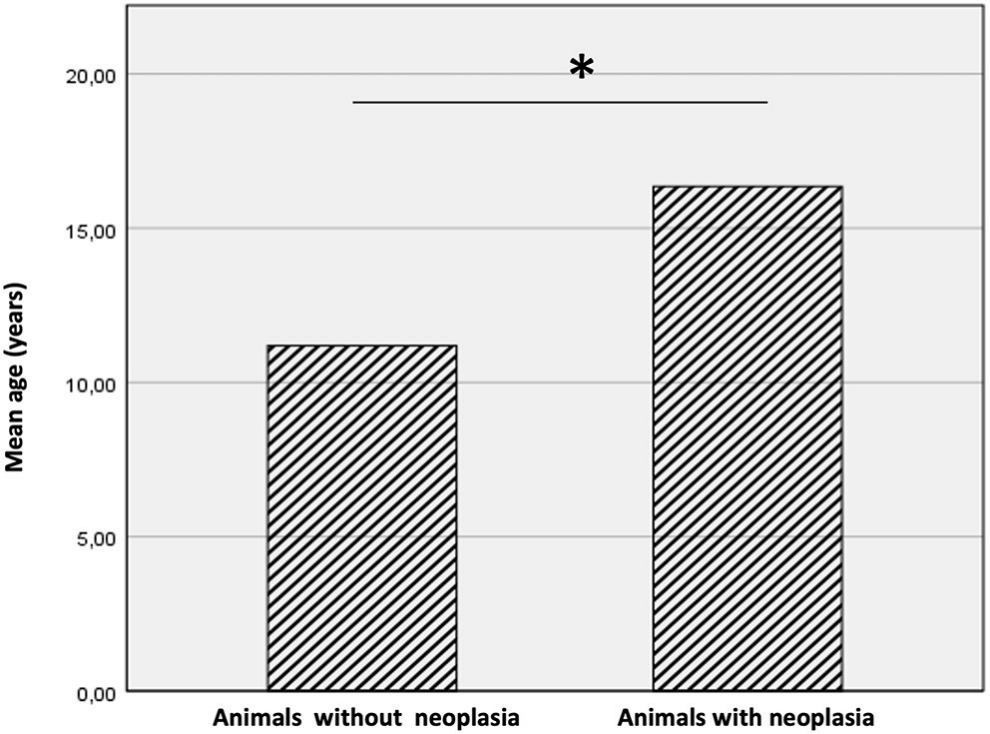
Mean age of animals with and without neoplasia. The animals with cancer were significantly older than animals without neoplasms. The * symbol indicates the value of *P* < 0.05.

A total of 24 cases fitted our inclusion criteria. Animals came from four different zoos in Southern Italy, 19 animals from Naples Zoo (Campania region), 2 animals from Maitine zoo in Benevento (Campania region), 2 animals from Fasano Zoo (Apulia region) and 1 animal from Aprilia Zoo (Lazio region). In particular, the species observed included 12 tigers (*Panthera tigris*), 7 leopards (*Panthera pardus*), 4 lions (*Panthera leo*), and 1 black jaguar (*Panthera onca*). The study population included 15 females and 9 males. Among the population, diagnosis of neoplasia was made in 14 out of 24 cases (58.3%) (NNR). Tumors occurred more frequently in tigers followed by leopards, lions, and black jaguar. The animals had an age range from 6 to 19 years; the mean age of all animals included in the study was 14.2 years (± 4.05 SD), the mean age of animals with neoplasia was 16.35 years (± 3.24 SD) while the mean age of animals without neoplasia was 11.2 years (± 3.08 SD). Animals with neoplasia were significantly older than animals without neoplasia (*P* < 0.05) (Figure 1). The frequency of neoplasia in female animals was 53.3% (8 of 15) while in male animals was 66.6% (6 of 9).

### Clinical and Pathological Findings

**Figure 2:**
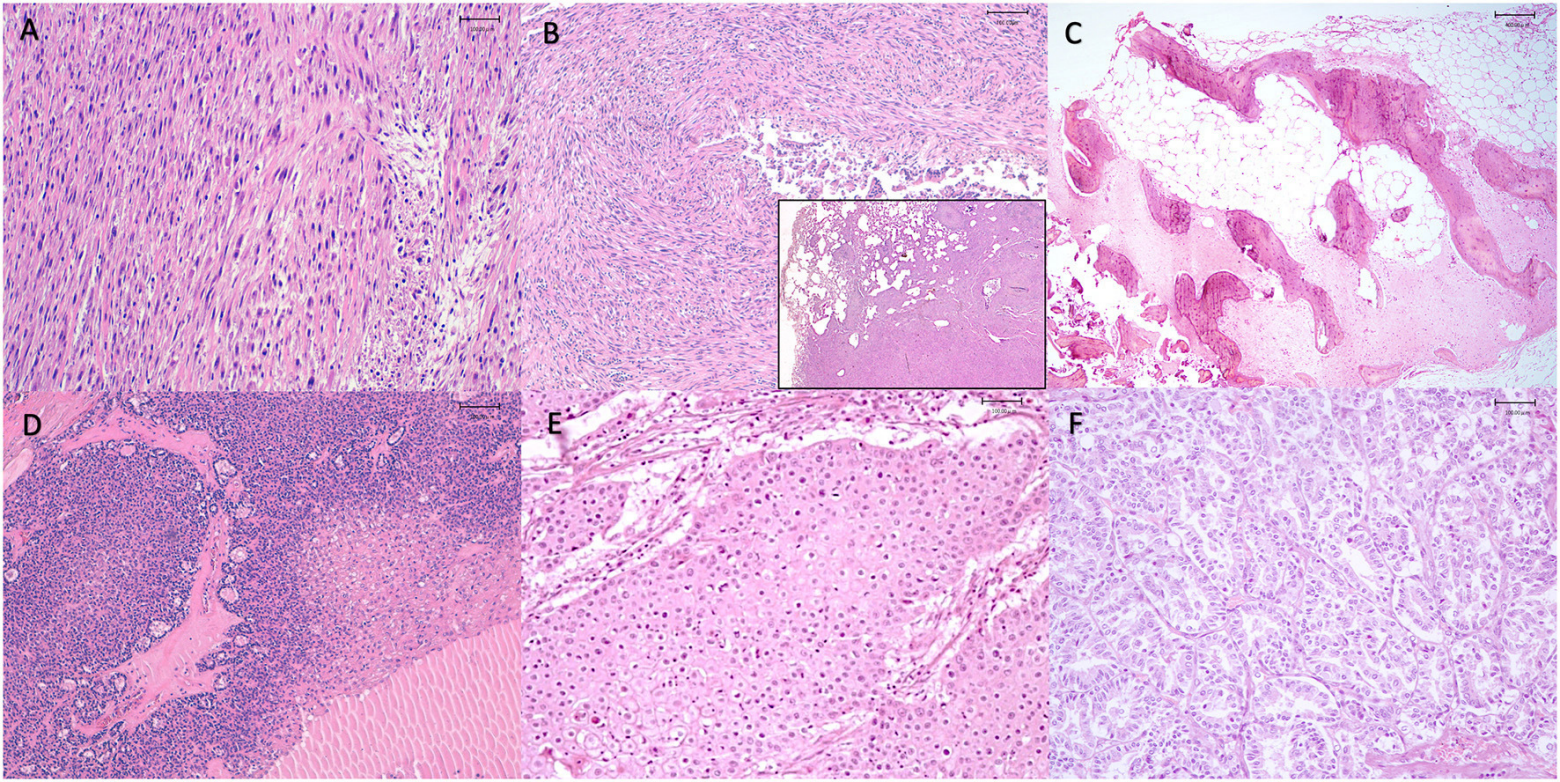
Representative neoplasms observed in captive wild felids in our study. **(A)** Spleen, metastatic squamous cell carcinoma, presence of numerous multiple round, white and firm nodules, Tiger (*Panthera tigris*), **(B)** Lungs, metastatic squamous cell carcinoma, presence of round, white and firm nodules, Tiger (*Panthera tigris*), **(C)** Uterus, leiomyoma, presence of multiple, white to yellow, firm masses, Lion (*Panthera leo*), **(D)** Liver, hemangiosarcoma, presence of multiple, red and white, soft masses that on cut surface appear mottled dark red and show an honeycomb appearance from tumor vascular spaces and abundant hemorrhage, Lion (*Panthera leo*), **(E)** Ribs, osteoma, presence of a solid, whitish mass extended from the 5th to the 7th rib, Tiger (*Panthera tigris*), **(F)** Liver, cholangiocarcinoma, presence of numerous red to white, coalescing cystic lesions, Leopard (*Panthera pardus*).

**Figure 3:**
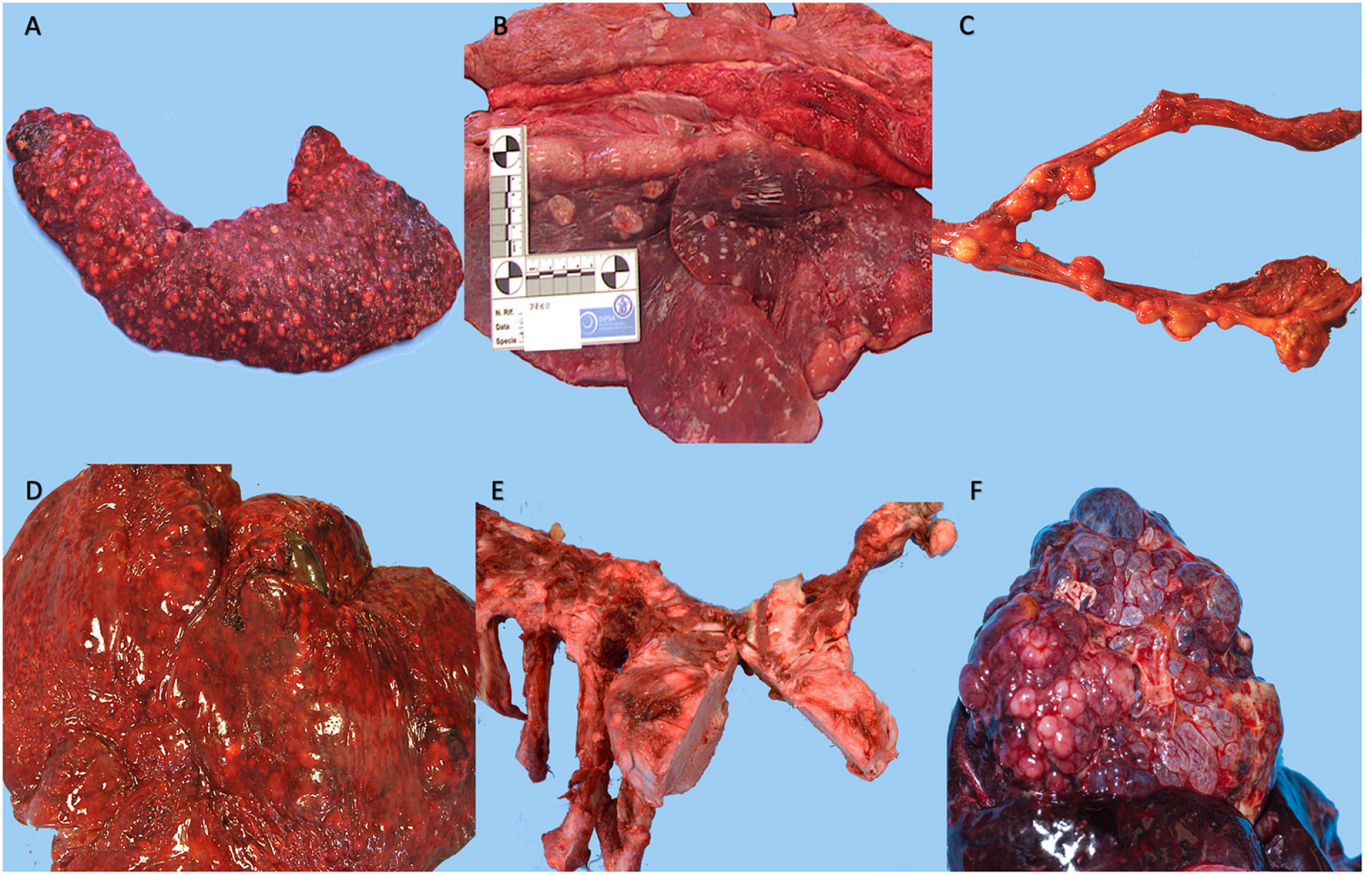
Representative histological section of tumors observed in our study. **(A)** Esophagus, leiomyosarcoma, neoplasm was composed of spindle-shaped cells arranged in streams, with brightly eosinophilic cytoplasm and oval nuclei (hematoxylin and eosin, 20x), Tiger *(Panthera tigris)*, **(B)** Lung, metastasis of leiomyosarcoma, expanding the pulmonary parenchyma (inset) there is a nodule composed of spindle-shaped cells arranged in streams, with brightly eosinophilic cytoplasm and oval nuclei (hematoxylin and eosin, 10x and 20x), Tiger *(Panthera tigris)*, **(C)** Rib, osteoma, neoplasm was composed of broad, interconnected bony trabeculae with central cores of woven bone surrounded by lamellar bone; intertrabecular spaces were filled with sparse spindle cells (hematoxylin and eosin, 4x), Tiger (*Panthera tigris*), **(D)** Thyroid, thyroid carcinoma, tumor cells were arranged in poorly-formed, variably-sized follicles filled with pale eosinophilic homogenous material (colloid), packets and solidly-cellular areas supported by a fine fibrovascular stroma. Areas of necrosis were observed, (hematoxylin and eosin, 20x) Tiger (Panthera tigris), **(E)** Soft palate, oral squamous cell carcinoma, neoplasm was composed of various sizes island and fronds of squamous cells with acid cytoplasm, pleomorphic and vesicular nuclei with prominent nucleoli, (hematoxylin and eosin, 20x), Tiger (Panthera tigris), **(F)** Liver, cholangiocarcinoma, neoplasm was composed of small, irregular, gland-like structures or packets of neoplastic cells embedded in connective tissue stroma. Cells had a moderate amount of clear to pale eosinophilic, round to oval and hyperchromatic nuclei with prominent nucleoli, (hematoxylin and eosin, 20x), Lion (*Panthera leo*).

Neoplasms were observed in the hepatobiliary (25%) (4 of 16), endocrine (18.7%) (3 of 16), reproductive (12.5%) (2 of 16), digestive (12.5%) (2 of 16), muscoloskeletal (12.5%) (2 of 16), urinary (6.2%) (1 of 16), respiratory (6.2%) (1 of 16), and nervous system (6.2%) (1 of 16) (Figure 2). Tumors diagnosed were as follow: two cholangiocarcinomas, two hemangiosarcomas of the liver, two uterine leiomyomas, a renal adenocarcinoma, an adrenal gland adenoma, a thyroid carcinoma, an oral squamous cell carcinoma, an osteoma, a meningioma, a mesothelioma, an esophageal leiomyosarcoma, a muscoloskeletal leiomyosarcoma and a thyroid adenoma (Figure 3). The frequency of epithelial tumors was 50% (8 of 16 tumors) while the frequency of mesenchymal tumors was 50% (8 of 16 tumors). Overall, 62.5% (10 of 16) of tumors were malignant and 37.5% (6 of 16) were benign. Among malignant tumors, no metastasis was observed in 50% of cases (5 of 10); in contrast, metastasis involving only regional lymph nodes were observed in 10% of cases (1 of 10) and distant metastases were found in 40% of cases (4 of 10). Most frequent observed clinical findings were ataxia, anorexia, weakness, and weight loss. Table 1 summarizes results about species, sex, age, organ system, clinical backgrounds, macroscopical and microscopical findings, type of neoplasia and cause of death.

**Table 1:** Summarized results about species, sex, age, organ system, clinical backgrounds, macroscopical and microscopical findings, type of neoplasia, and cause of death.

**Species**	**Sex**	**Age**	**System**	**Clinical data**	**Macroscopic findings**	**Histopathological findings**	**Neoplasia**	**Cause of death**
Lion (Panthera leo)	M	19	Hepatobiliary system	Weakness, anorexia, weight loss.	Multifocal to coalescing, non-encapsulated, yellowish and firm nodules with an umbilicated appearance ranging from 3 to 6 cm in diameter, distributed throughout the liver lobes. On the cut surface the nodules showed an infiltrative growth pattern with frequent central necrosis.	Neoplasm was composed of small, irregular, gland-like structures or packets of neoplastic cells embedded in connective tissue stroma. Cells had a moderate amount of clear to pale eosinophilic, often finely granular cytoplasm, round to oval and hyperchromatic nuclei with prominent nucleoli. Mitotic count was 40 per 10 HPFs.	Cholangiocarcinoma	Spontaneous
Lion (Panthera leo)	F	17	Hepatobiliary system; genital system	Lethargy, anorexia, hematemesis, and difficulty walking	Multiple, red and white, soft masses were observed in the liver; on cut surface they appear mottled dark red and show an honeycomb appearance from tumor vascular spaces and abundant hemorrhage. Uterus: Multiple, white to yellow, firm masses were observed.	Liver: neoplasm was composed of endothelial cells that lined spaces in mostly a single layer and hyperchromatic, plump and larger than normal endothelial cells. Anisokaryosis, anisocytosis, prominent nucleoli and hyperchromasia were observed. Mitotic count was 25 per 10 HPFs. Uterus: neoplasm was characterized by variably sized interlacing fascicles of well-differentiated smooth muscle cells with rare mitotic figures. Cells were characterized by indistinguishable eosinophilic cytoplasm and “cigar-shaped” nuclei.	Hemangiosarcoma of the liver; Uterine leiomyoma	Spontaneous
Jaguar (Panthera onca)	F	15	Hepatobiliary system	Ataxia, anorexia, weakness.	Multiple, red and whitish nodules with central necrosis were observed in the liver; some masses contained cystic structures.	Expanding the liver was a poorly circumscribed, moderately cellular, infiltrative neoplasm composed of spindle cells forming irregularly sized blood-filled vascular channels. Neoplastic cells had variably distinct cell borders, moderate amount of eosinophilic fibrillar cytoplasm, irregularly oval to elongate nuclei, coarsely clumped chromatin and up to 2 distinct nucleoli. Anisokaryosis and anisocytosis were marked. Mitotic count was 20 per 10 HPFs.	Hemangiosarcoma of the liver.	Spontaneous
Leopard (Panthera pardus)	M	17	Hepatobiliary system	Two weeks of starvation, lethargy and weakness.	Size of the liver was increased and numerous red to white, coalescing cystic lesions were found.	Neoplasm was composed of polygonal cells arranged in irregular, branching, and anastomosing tubules, and acini on a moderate fibrovascular and desmoplastic stroma. Neoplastic cells had variably distinct cell borders, a moderate amount of pale eosinophilic and vacuolated cytoplasm, one round to oval nucleus, with finely-stippled chromatin and 1–2 distinct basophilic nucleoli. The mitotic rate was 15 per 10 HPFs.	Cholangiocarcinoma	Spontaneous
Leopard (Panthera pardus)	M	18	Urinary system; endocrine system.	Long history of limp, died after a few weeks of starvation and lethargy. Hematological and biochemical exams revealed severe anemia, hyperazotemia and liver enzymes alteration.	The right kidney was indiscernible and fuse with the liver. Liver was invaded by several, variable in size neoplastic masses (2–5 cm in diameter). A well-circumscribed and whitish mass was observed in the left lobe of thyroid gland.	Kidney: neoplasm was composed of a densely cellular neoplastic population of polygonal cells arranged in tubules or island and separated by fine strands of fibrovascular stroma. Neoplastic cells had distinct cell borders, small amounts of finely granular, eosinophilic cytoplasm and a well-distinguishable border. The nuclei were irregularly round, with finely stipule chromatin and 1–2 variably distinct nucleoli. Mitosis werw 10 per 10 HPFs). Metastatic cells were observed in the liver. Thyroid: Neoplastic cells formed irregularly shaped follicles with occasional papillary infoldings of epithelium and variable amounts of colloid. Mitotic figures were 10 per 10 HPFs.	Renal adenocarcinoma; Thyroid Adenoma.	Spontaneous
Leopard (Panthera pardus)	F	17	Genital system	Persistent estrus signs. Ultrasound exam revealed an enlarged size of ovaries and the presence of ovarian cysts.	Uterus examination revealed a solid and yellowish mass of 5 cm in diameters.	Neoplasm was characterized by variably sized interlacing fascicles of well-differentiated smooth muscle cells with rare mitotic figures. Cells were characterized by indistinguishable eosinophilic cytoplasm and “cigar-shaped” nuclei.	Leiomyoma	Spontaneous
Tiger (Panthera tigris)	F	17	Endocrine system	Muscle weakness and fatigue.	The cortex of the adrenal gland showed a well-demarcated and yellow nodule. Adjacent cortical parenchyma was compressed.	Neoplasia was capsulated; tumor cells were arranged in trabeculae or nests separated by small vascular spaces. Cells were characterized by abundant, lightly eosinophilic, and often vacuolated, cytoplasm. Mitotic count was 2 per 10 HPFs.	Adrenal adenoma	Euthanasia
Tiger (Panthera tigris)	F	18	Digestive system	Severe weight loss and a neck wound with discharging sinus tracts. Hematological and biochemical analysis indicated an alteration of hepatic parameters and anemia.	A large mass of esophagus was observed. Several yellowish and whitish lungs nodules were observed, consistent with metastasis of esophageal mass.	Neoplasm was composed of spindle cell beams intertwined in various directions. Cell borders were indiscernible, cytoplasm was moderate to abundant and distinctly eosinophilic. Cells had oval nuclei, sometimes blistered with dispersed chromatin. Mitotic figures were 20 per 10 HPFs. Neoplastic cells were strongly immunopositive to vimentin (DAKO, clone 9) and to α-smooth muscle actin (DAKO, clone 1A4).	Leiomyosarcoma	Spontaneous
Tiger (Panthera tigris)	F	18	Endocrine system	Animal showed neurologic signs, such as convulsions and ataxia. Afer an improvement of its conditions thanks to steroids treatment, the tiger showed weakness and weight loss and was euthanized.	A round, capsulated, light brown neoplasm was observed in the thyroid. Fibrosis, hemorrhage, and cystic changes were also observed.	Tumor cells were arranged in poorly-formed, variably-sized follicles filled with pale eosinophilic homogenous material (colloid), packets and solidly-cellular areas supported by a fine fibrovascular stroma. Neoplastic cells had indistinct cell borders, a moderate amount of eosinophilic vacuolated cytoplasm, one irregularly round to ovoid nucleus with finely-stippled chromatin and one to two distinct nucleoli. Anisokaryosis and anisocytosis were moderate. Areas of necrosis were observed. Mitotic count was 15 in 10 HPFs. Same neoplastic cells were observed in the regional lymph node.	Thyroid Carcinoma	Euthanasia
Tiger (Panthera tigris)	F	18	Digestive system	Found dead after 2 weeks of respiratory disorders, rhinorrhea and lethargy.	A poorly demarcated and infiltrated mass of the soft palate, involving the adjacent tissues, was observed. Mandibular and prescapular lymph nodes were enlarged and in cut surface multiple firm and grayish nodules were found. The spleen revealed the presence of round, firm and white nodules. Multiple whitish masses were also observed in the liver and in the lungs.	The mass of the soft palate was composed of various sizes island and fronds of squamous cells with acid cytoplasm, pleomorphic and vesicular nuclei with prominent nucleoli, increased nuclear cytoplasm ratio and numerous mitotic figures (30 per 10 HPFs). Same neoplastic cells were observed in the spleen and in the lung.	Squamous cell carcinoma	Spontaneous
Tiger (Panthera tigris)	M	17	Musculoskeletal system	Lameness, weakness and starvation. Left forelimb lameness was treated with anti-inflammatory drugs with no improvement.	A solid, whitish mass extended from the 5th to the 7th rib was found.	Neoplasm was composed of broad, interconnected bony trabeculae with central cores of woven bone surrounded by lamellar bone. Intertrabecular spaces were filled with sparse spindle cells. Mitotic count was 1 per 10 HPFs.	Osteoma	Euthanasia
Tiger (Panthera tigris)	M	14	Central nervous system	Stuporous state and death few hours after symptoms occured.	A soft, well-demarcated mass arising from the meninges was seen in the prefrontal cortex.	A well-demarcated, densely cellular neoplasm composed of meningothelial cells that emanated from meningeal tissues, formed tightly packed whorls and spiraled streams in a fibrovascular stroma. Mitotic count was 5 per 10 HPFs. Neoplastic cells were strongly immunopositive to vimentin (DAKO, clone 9) while synaptophysin (DAKO) was uniformly negative. Moreover, immunopositivity to S-100 (DAKO) and cytokeratin (DAKO, Clone AE1/AE3) were focal.	Meningioma	Spontaneous
Tiger (Panthera tigris)	F	6	Musculoskeletal system	Lameness, weakness and anorexia.	An infiltrative, 20 cm in diameter, not capsuled mass arising from the muscle of the forelimb was observed. Central necrosis was observed.	Neoplasia was formed by densely packed spindle cells. Cells were ovoid or round, with abundant eosinophilic cytoplasm and elongated nuclei with granular chromatin. Mitotic count was 15 per 10 HPFs. Necrosis was observed. Neoplastic cells were strongly immunopositive to vimentin (DAKO, clone 9) and to α-smooth muscle actin (DAKO, clone 1A4).	Leiomyosarcoma	Euthanasia
Tiger (Panthera tigris)	M	18	Pleura	Cachexia, anorexia.	Variably sized, discrete, or confluent, firm, white masses scattered throughout the pleural surfaces of the thoracic wall and of the lungs were observed.	Neoplasm consisted of neoplastic cells arranged in branching outgrowths supported by a central stromal core. The neoplastic cells were monomorphic and well-differentiated. Papillae were lined by a single layer of neoplastic mesothelial cells within large areas of the neoplasm. Mitotic count was 5 in 10 HPFs.	Epithelioid papillary mesothelioma.	Euthanasia

*In this table are summarized results about species, sex, age, organ system, clinical backgrounds, macroscopical and microscopical findings, type of neoplasia and cause of death*.

## Discussion

The present study aimed to evaluate the frequency of neoplasms in captive wild felids housed in Southern Italy Zoos during a period of 13 years (2008-2021) and to investigate clinical, macroscopic and histologic tumor findings in these animals. The rate of neoplasia in our studied population (58.3%) is higher compared to studies carried out in other zoological gardens, such as Philadelphia and Knoxville zoological gardens. In these studies, rates of neoplasia were much lower and varied from 2.6 to 51% (9, 15). Similarly, a rate of neoplasia of 50.2% was observed in the studied population analyzed by the Anatomic Pathology Service of the University of Tennessee Veterinary Medical Center (11). Differences between studies could be due to a broad range of intrinsic and extrinsic variables, such as environmental factors, population size, and animal longevity. Indeed, zoos included in our study are located in the city center; Naples, in particular, is the third most populated municipality in Italy characterized by many environmental stressors, deriving from intensive car traffic and widespread industrial activities (16). Prolonged exposure to these stressors is indeed associated with an increased risk of developing cancer in animals as well as humans (1). Our study showed a mean age of animals with neoplasia at necropsy of 16.35 (± 3.24 SD), significantly higher when compared to animals without neoplasia (11.2 ± 3.08 SD) and overall higher than those reported in previously published papers. These findings can be explained considering that cancer is an age-related disease with a higher risk in old animals (17). The most common tumors observed were cholangiocarcinoma, hemangiosarcoma, uterine leiomyoma, leiomyosarcoma followed by a renal adenocarcinoma, an adrenal gland adenoma, a thyroid carcinoma, an oral squamous cell carcinoma, a meningioma, a thyroid adenoma and an osteoma.

Liver was the organ most commonly affected by neoplasia (4/16, 25%); in contrast, primary hepatic tumors are relatively uncommon in domestic animals; however, in cats, cholangiocellular carcinomas are reported to be the most frequent primary hepatic malignancy (18). Among hepatic tumors, we observed two cases of cholangiocarcinoma, one in a male lion and one in a male leopard, and two cases of hemangiosarcoma in a female lion and in a female jaguar. In human medicine, several risk factors have been shown to be important in the development of cholangiocarcinomas such as fluke infestation, inflammation, and chronic injury of the biliary epithelium (19). However, no predisposing factors were identified in these cases. Hemangiosarcoma is very rare in domestic cats as well as in large captive felids (20). Primary hemangiosarcoma of the liver has been rarely described in wild captive felids (21).

An oral SCC was observed in a female lion; SCC is the most common oral neoplasm in domestic cats (22). Although the most frequent site of SCC in non-domestic animals is the skin (9, 23–25), oral squamous cell carcinoma has been recently reported in captive felids (26–29). Cutaneous SCCs are associated with prolonged exposure to ultraviolet light, lack of pigment in the epidermis, lack of hair, and infection with Felis catus papillomavirus type-2 (FcaPV-2) (22). The causes of oral SCC in cats are still unclear; although in humans it has been associated with tobacco smoke and papillomavirus, there is no evidence of these associations in cats. Feline oral SCC is highly locally invasive, but metastasis occurs infrequently and usually to regional lymph nodes. In our study, we report for the first time, to the best of our knowledge, a case of SCC in a female tiger with metastasis in the lungs and spleen.

Endocrine neoplasms were reported in two felids, a thyroid carcinoma in an 18-year-old female tiger and a thyroid adenoma in an 18-year-old male leopard. Thyroid tumors are commonly diagnosed in captive felids and have been previously reported in several studies (9, 11, 15, 30–32). This low rate of endocrine tumors is, in fact, in contrast with previous studies (9, 30). Pathogenetically, an iodine deficiency presumably due to a deficit of the iodine supplementation was associated with a higher incidence of these endocrine tumors (15). In the present study, no data about iodine supplementation was found and it remains undetermined whether similar factors have influenced the development of thyroid neoplasms.

Tumors of the reproductive system occurred in a female lion and in a female leopard and were represented by two uterine leiomyomas. Leiomyomas are the most common mesenchymal tumor type in the uterus and are commonly diagnosed in large felids (9, 11).

Tumors of the nervous system are rarely described in captive wild felids, among these a meningioma was reported in a bengal tiger (33). Meningiomas are tumors that arise from the meningeal cells and are the most frequently reported intracranial tumor of the cat (60%) (33). We report a case of a meningioma in a 14-year-old male tiger that showed a stuporous state and died a few days after the symptom appearance; at the necropsy a soft mass arising from the meninges was seen in the prefrontal cortex. Immunohistochemistry studies were performed in order to better characterize the lesion (33, 34).

A mesothelioma was diagnosed in the pleura of a male tiger; mesothelioma is a neoplasm that arises from the mesothelium, the layer of cells that lines the serous cavities of the body: pleura, peritoneum, pericardium, etc. Despite their rarity in animals, pleural mesotheliomas have been already reported in tigers (6).

This study identified some neoplasms not previously or rarely described in wild captive felids. Of those, one esophageal leiomyosarcoma was found in a female tiger and one leiomyosarcoma of the skeletal muscle was diagnosed in a female young tiger. Esophageal neoplasia is very rare in domestic and wildlife animals (35, 36), and only one case of an esophageal angioleiomyosarcoma in a cat was reported in the literature (36). Other previously unreported neoplasms in captive felids included an osteoma in a male tiger, an adrenal adenoma in a female tiger, and a renal adenocarcinoma in a male leopard.

## Conclusion

This study established frequency rates, primary tumor site and tumor types in captive wild felids in Southern Italy Zoos over a 13-year period. Based on our findings, the liver was the most primary frequent tumor site. Cholangiocarcinoma, hemangiosarcoma and leiomyosarcoma were the most common tumors identified. The high rates of malignant and widely metastatic neoplasms suggest the importance of active monitoring and management of neoplasia in these threatened and endangered species. Further research about the role of environment, genetics, and aging is necessary to shed some light on the risk factors for the development of tumors in wild captive felids.

## Data Availability Statement

The raw data supporting the conclusions of this article will be made available by the authors, without undue reservation.

## Ethics Statement

Ethical review and approval was not required for the animal study because the study did not require consent or ethical approval according to D.lgs 21 marzo 2005, n. 73. Necropsies were performed on cadavers for diagnostic purpose and permission to obtain samples was granted from the veterinaries of the zoos responsible for the sanitary surveillance and owners of the zoos.

## Author Contributions

Id'A drafted the manuscript and contributed to the study concept, study design, analysis, and interpretation of data. Id'A, GP, FP, SC, BD, OP, and VR conducted the necropsies and the histopathological analysis. Id'A, GP, SC, PS, GR, MC, BD, PL, OP, and VR revised the manuscript for content and contributed to the interpretation of data. OP and VR supervised all study and also contributed to the study concept and design. All authors have read and agreed to the published version of the manuscript.

## Conflict of Interest

The authors declare that the research was conducted in the absence of any commercial or financial relationships that could be construed as a potential conflict of interest.

## Publisher's Note

All claims expressed in this article are solely those of the authors and do not necessarily represent those of their affiliated organizations, or those of the publisher, the editors and the reviewers. Any product that may be evaluated in this article, or claim that may be made by its manufacturer, is not guaranteed or endorsed by the publisher.
